# Enhanced MCM5 Level Predicts Bad Prognosis in Acute Myeloid Leukemia

**DOI:** 10.1007/s12033-022-00623-9

**Published:** 2022-12-07

**Authors:** Shuming Wang, Weiqin Wu, Xiang Han

**Affiliations:** 1 Department of Pediatric General Surgery, The Affiliated Huaian No.1 People’s Hospital of Nanjing Medical University, Huai’an, Jiangsu China; 2 Department of Emergency, The Affiliated Huaian No.1 People’s Hospital of Nanjing Medical University, Huai’an, Jiangsu China

**Keywords:** DNA replication, MCM5, Acute myeloid leukemia, Prognostic marker, Prognosis

## Abstract

Acute myeloid leukemia (AML) is a fatal heterogeneous hematologic malignancy. There is an urgent need to identify potential biomarkers to better classify sufferers with bad outcomes that might need more advanced treatment. The objective of this study was to investigate prognostic indicators that predict the outcome of sufferers with AML. The datasets of AML sufferers including mRNA sequencing data and clinical information were acquired from GEO datasets (GSE38865) and TCGA datasets. Kaplan–Meier curves and Cox regression analysis to screen genes correlated to survival. Gene Ontology (GO) and Kyoto Encyclopedia of Genes and Genomes (KEGG) analyses biological process analysis were utilized in verifying the function of various genes. Sufferers with elevated MCM5 level exhibited a worse prognosis, according to the survival analysis. It was indicated through multivariate and univariate analysis that MCM5 level was an independent adverse prognostic element for over survival in AML sufferers based on GEO and TCGA datasets. Meanwhile, MCM5 level in AML samples was higher than in normal samples. Additionally, it was indicated through PPI network and functional enrichment analyses that through accelerating cell cycle and DNA replication, MCM5 promoted AML progression. In conclusions, MCM5 level was an independent poor prognostic element in AML sufferers based on GEO and TCGA datasets. This is the first time that MCM5 is reported to be a biomarker of poor prognosis in AML.

**Supplementary Information:**

The online version contains supplementary material available at 10.1007/s12033-022-00623-9.

## Background

In adults, the most common acute leukemia is acute myeloid leukemia (AML) [1]. AML is a cell clonal malignant proliferative disease of myeloid primordial cells in the hematopoietic system. AML is a highly heterogeneous group of diseases that can be derived from hematopoietic progenitors at various stages of differentiation and development of normal myeloid cells [2–4]. The incidence of AML increased proportionally with age, from 1.8 cases per 100,000 people under 65 years old to 13.7 cases per 100,000 people over 65 years old. In developed countries, various half of novelly diagnosed AML sufferers are over 65 years, with a median age of 67 years at diagnosis, and more men have AML than women [5]. There are various 20,000 new AML cases in the US in 2021 [6]. On the other hand, merely about 25% of AML sufferers survived for 5 years or more despite multi-drug combination chemotherapy. Elderly sufferers (> 60 years) and sufferers that could not bear standard induction chemotherapy had unfavorable molecular outcomes, with a median survival of merely 5–10 months and a 5-year overall survival (OS) of 5% [7, 8]. At present, scientific research has moved from the cellular level to the molecular level. For example, targeted drugs [9], nanomaterials [10], and molecular markers [11]. We are committed to finding new molecular markers to more accurately predict the progress and prognosis of AML.

Microchromosome maintenance protein 5 (MCM5) is a key cell cycle regulator located on chromosome 22Q13.1 [12], whose role in DNA replication is verified [13]. MCM5 is a DNA licensing factor as an ingredient of the MCM2-7 complex, a putative replicative helicase vital for the initiation and extension of "once per cell cycle" DNA replication in eukaryotic cells [14, 15]. So far, MCM5 has been reported to be closely correlated with varieties of diseases. For instance, increased level of MCM5 is remarkably correlated with the positive progression and unfavorable prognosis of oral squamous cell carcinoma, and MCM5 can be utilized as a marker for the early diagnosis of oral squamous cell carcinoma [16, 17]. MCM5 was also reported to be an individual prognostic element in lung squamous cell carcinoma [18]***.*** MCM5 can aggravate the HDAC1-mediated malignant progression of lung cancer [19]. MCM5 is correlated with malignant status and unfavorable prognosis in cervical adenocarcinoma sufferers, and regulates the proliferation of cervical adenocarcinoma cells [20]. MCM5 is a new sensitive as well as specific biomarker for the detection of endometrial and ovarian tumors in urine samples [21]. Therefore, we investigated the relationship betwixt MCM5 and AML.

In this study, we investigated the gene level microarray of AML and correlative clinical data in GSE38865, GSE142698, and The Cancer Genome Atlas (TCGA) database. MCM5 was selected as the research objective. Then, we studied the level profile and biological functions of MCM5 in AML, and further analyzed the correlation betwixt MCM5 level and the AML.

## Methods

### Data acquisition

With gene expression omnibus (GEO, https://www.ncbi.nlm.nih.gov/geo/) database, “AML” and “survival” in the search box as the keyword, we harvested the AML gene level chips and correlative clinical data. GSE38865 [22] was selected as the training data set of this study. We extracted clinical data with prognostic information directly from the matrix file on the correlative gene chip page in the GEO database.

From TCGA (https://portal.gdc.cancer.gov/) we harvested RNA-seq data (TPM) and the correlative clinical information of AML.

### Screening of Prognosis-Related Genes

Kaplan–Meier curves and Cox regression analysis to screen genes correlated to survival. The prognosis-related genes screened through “survival” R package with the KM < 0.01 and cox *p* value < 0.01 were shown in Table S1.

### Identify Target Gene

After screening of prognosis-related genes, we utilized multivariate Cox regression analysis to perform individual prognostic analysis (*p* < 0.01). Thus, we got the genes remarkably correlated with individual prognosis which were exhibited in Table 1.

**Table 1:** The genes for independent prognostic selected by multivariate Cox regression analysis (*p* < 0.01)

id	HR	HR.95L	HR.95H	pvalue
ABCB9	19.45033	3.316165	114.0822	0.001009
ACAA1	0.032734	0.004066	0.263502	0.001312
AEBP1	2.782877	1.34452	5.759978	0.005823
ALS2CR4	6.917057	1.803126	26.53485	0.004812
ARF5	0.008739	0.000465	0.164135	0.001537
ARHGAP26	0.168013	0.048536	0.581596	0.004871
BRP44L	0.069428	0.012614	0.382125	0.002173
C18orf10	257.4444	9.680502	6846.509	0.000913
CYP2C19	57.22527	2.775849	1179.723	0.008761
DDAH1	26.18649	2.935991	233.5607	0.003448
DENND4C	0.013585	0.000848	0.217521	0.002381
DKC1	27.41135	3.020252	248.7813	0.003259
DKFZp761P0423	0.136307	0.040676	0.45677	0.001238
DOCK10	0.235632	0.078794	0.704652	0.009702
DYNC1I1	54.32938	4.393111	671.8887	0.00185
ELOVL7	3.715408	1.512775	9.125119	0.004198
EXTL3	0.065921	0.010681	0.40686	0.003407
FAM92A1	3.263457	1.402455	7.593934	0.006053
FLII	0.021347	0.001606	0.283668	0.003562
GGH	5.791323	1.691516	19.82802	0.005157
KL	10.06271	1.811927	55.88421	0.008303
KLF4	0.16852	0.054797	0.518265	0.001892
KNTC1	21.57496	3.036184	153.3105	0.00214
LAPTM4B	2.555265	1.294228	5.045001	0.00687
LOC389599	14.1511	2.751923	72.76859	0.001516
LOC642756	0.00273	7.31E-05	0.101963	0.001393
LOC645227	0.002868	5.41E-05	0.152084	0.003858
LOC646596	0.03238	0.002995	0.350019	0.004738
MCM5	10.25121	1.935775	54.28697	0.006208
NEDD4	36.96468	4.100256	333.2445	0.001292
NEK3	2.04E-08	6.06E-13	0.000687	0.000871
NLRP1	0.067779	0.010207	0.450079	0.005329
NUDT21	53.38075	3.753781	759.1024	0.003319
OR1J2	0.019879	0.00115	0.343559	0.007043
PAQR3	13.02457	2.444484	69.3968	0.002637
PDLIM1	2.547623	1.273733	5.09556	0.008192
PFKM	4.841262	1.68165	13.93739	0.003462
PITPNC1	0.141614	0.03599	0.557233	0.005164
PLOD3	0.162967	0.048746	0.544821	0.003217
PNMA1	14.78089	3.205908	68.14751	0.000552
PRRT3	2.756331	1.279435	5.938059	0.009618
REXO2	48.614	4.244601	556.7829	0.001796
RFC4	16.2703	2.922564	90.57884	0.001451
SEZ6L2	14.28327	1.959829	104.0968	0.008692
SIAH2	259.6796	7.06392	9546.184	0.002503
SLC40A1	9.944357	2.157703	45.83125	0.003215
SNRPE	13.15298	1.863505	92.83628	0.009759
TMEM38B	10.7152	2.379157	48.25887	0.00201
TXNDC9	10,593,500	66.96155	1.68E + 12	0.008091
ZFP1	30.16476	3.232077	281.5258	0.002795
ZWILCH	33.12339	3.038568	361.0777	0.004081
ZYX	0.005623	0.000182	0.17335	0.003059

Next, we analyze the correlation betwixt individual prognostic-related genes and clinical traits through Wilcox.test or Kruskal.test (*p* < 0.05). The correlation betwixt individual prognostic-related genes and clinical traits was shown in Table 2.

**Table 2:** The clinical traits related-independent prognostic genes

ID	Gender	Age	Wbc	NPM1 mutation	SigNum
DENND4C	0.046452	0.046452	0.080824	0.069096	2
DKC1	0.038128	0.602679	0.528388	0.115266	1
LAPTM4B	0.111943	0.766432	0.25409	0.047345	1
MCM5	0.602679	0.940843	0.04002	0.472668	1

Then, we utilized GEPIA (http://gepia2.cancer-pku.cn) to explore the relationship betwixt genes in Table 2 (SigNum > 0) and the survival of AML.GEPIA is an interactive web server [23].

### Survival Analysis

All AML sufferers with MCM5 level values higher than the median were classified as MCM5 high group, and the remaining sufferers were classified as MCM5 low group. We utilized Kaplan–Meier curve and log-rank test via the R package “survival” and “survminer” to analyze the survival of MCM5 in the prognostic model. Individual prognostic value of MCM5 was verified with multivariate and univariate Cox regression analyses.

### Enrichment Analysis

The difference of gene level in AML samples betwixt MCM5 high group and MCM5 low group was compared through setting adjusted *p* < 0.05 and fold change > 1 threshold using the ‘‘Limma” package. "ClusterProfiler" and "enrichplot" packages were utilized for Kyoto encyclopedia of genes and genomes (KEGG) analysis and Gene Ontology (GO) to further explore the functions of various genes.

### Statistical Analysis

Through R software 3.5.0 we performed all statistical analysis. We utilized Fisher exact test as well as the Wilcoxon rank-sum tests, respectively, to verify hypotheses for categorical and continuous variables. According to the median level value of MCM5, the samples in the second cohort were divided into MCM5 high group (*n* = 10) and MCM5 low group (*n* = 10). The limma package was utilized to analyze the distinct gene level. Kaplan–Meier method and Cox regression multivariate analysis were utilized for survival analysis, and log-rank test was utilized for comparison betwixt groups. To identify GO and KEGG enrichment terms, we utilized “Clusterprofiler” package. For all statistical analysis, *p* < 0.05 was considered significant.

## Results

### Screening Target Genes

We got GSE38865 from GEO database (https://www.ncbi.nlm.nih.gov/geo/). According to the probe information of Illumina HumanWG-6 V3.0 expression beadchip (GPL6884) and Illumina HumanHT-12 V4.0 expression beadchip (GPL10558), 22089 genes of 30 AML samples were annotated. Then, we performed Kaplan–Meier curves and Cox regression analysis to screen genes correlated to survival. The prognosis-related genes were screened through “survival” R package with the KM < 0.01 and coxPvalue < 0.01 (Table S1).

In order to get the genes for independent prognostic, multivariate Cox regression analysis was performed (*p* < 0.01) in prognosis-related genes. 52 genes for independent prognostic were exhibited in Table 1.

Later, we analyze the correlation betwixt individual prognostic genes and clinical traits (age, gender, Wbc, and NPM1 mutation) through wilcox.test or kruskal.test (*p* < 0.05). Four clinical traits related-individual prognostic genes (DENND4C, DKC1, LAPTM4B, and MCM5) were shown in Table 2.

**Fig. 1:**
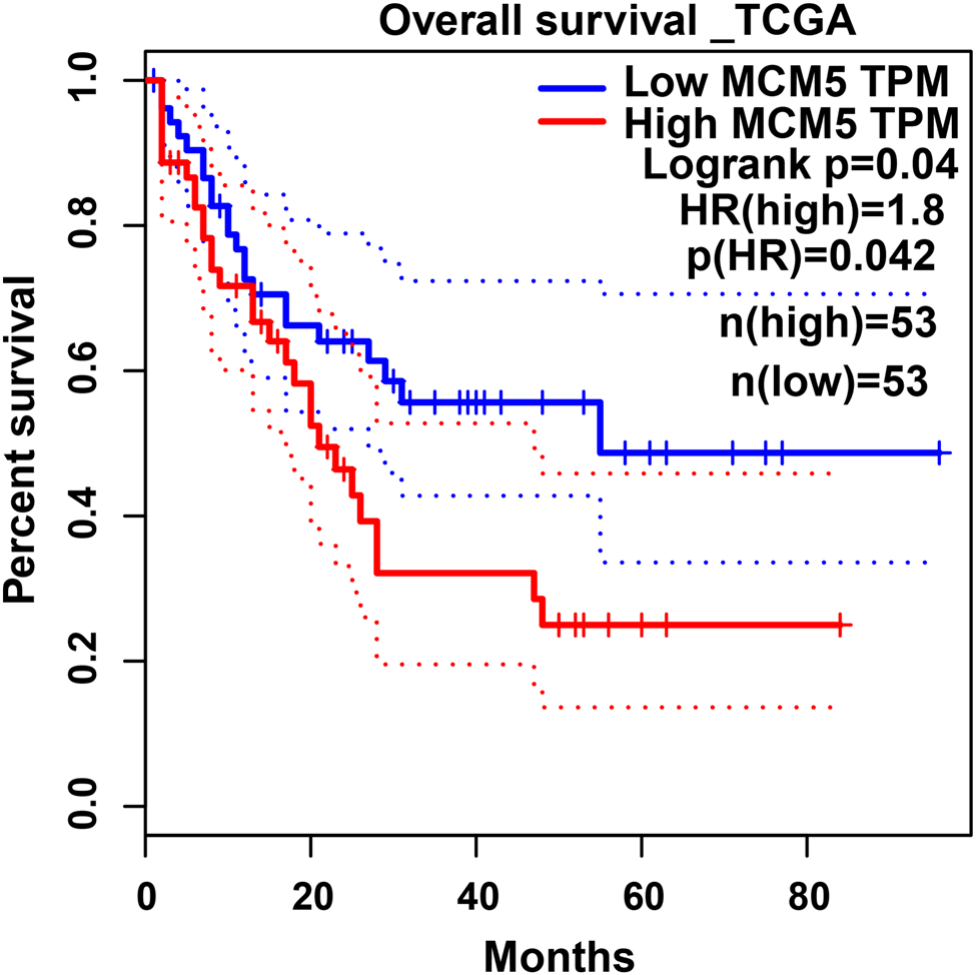
Survival outcomes in MCM5 high group and MCM5 low groups through GEPIA. AML sufferers in MCM5 high group had worse outcome than that in MCM5 low group

GEPIA (http://gepia2.cancer-pku.cn) was utilized to verify the relationship betwixt these 4 genes and the over survival of AML. We found that merely MCM5 was correlated to the survival and prognosis of AML. As presented in Fig. 1, sufferers with high level of MCM5 had worse prognosis than these with low level of MCM5. Therefore, we chose MCM5 for follow-up study.

### Identify Target Gene MCM5

**Fig. 2:**
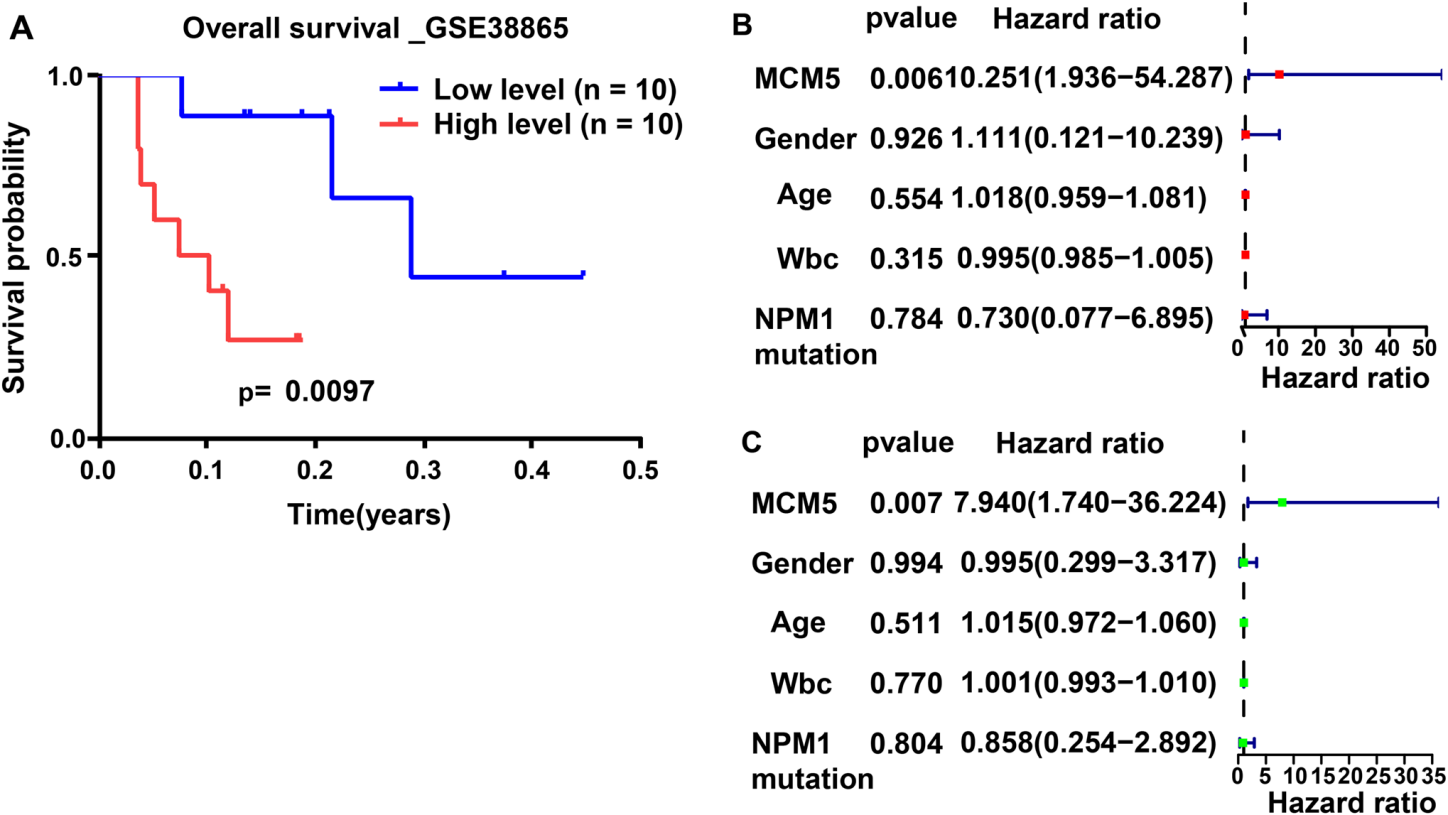
Survival analysis and Cox regression analysis of MCM5 in GSE38865. **A** Survival analysis betwixt MCM5 high group and MCM5 low groups in GSE38865. **B** Univariate Cox regression analysis and **C** multivariate Cox regression analysis screened out the individual prognostic-related element

In GSE38865, all AML sufferers with MCM5 level values above the median were classified as MCM5 high group, and the others were considered to be MCM5 low group. As shown in Fig. 2A, the prognosis of AML sufferers with high MCM5 level was worse than that of sufferers with low MCM5 level. Multivariate and univariate Cox regression analysis exhibited that MCM5 was remarkably correlated with the prognosis of AML sufferers and was an independent prognostic element. (Fig. 2B and C; Table S2).

### MCM5 in TCGA database

To further identify target gene MCM5, we harvested RNA-seq data (TPM) and the correlative clinical information from TCGA-AML (https://portal.gdc.cancer.gov/).

**Fig. 3:**
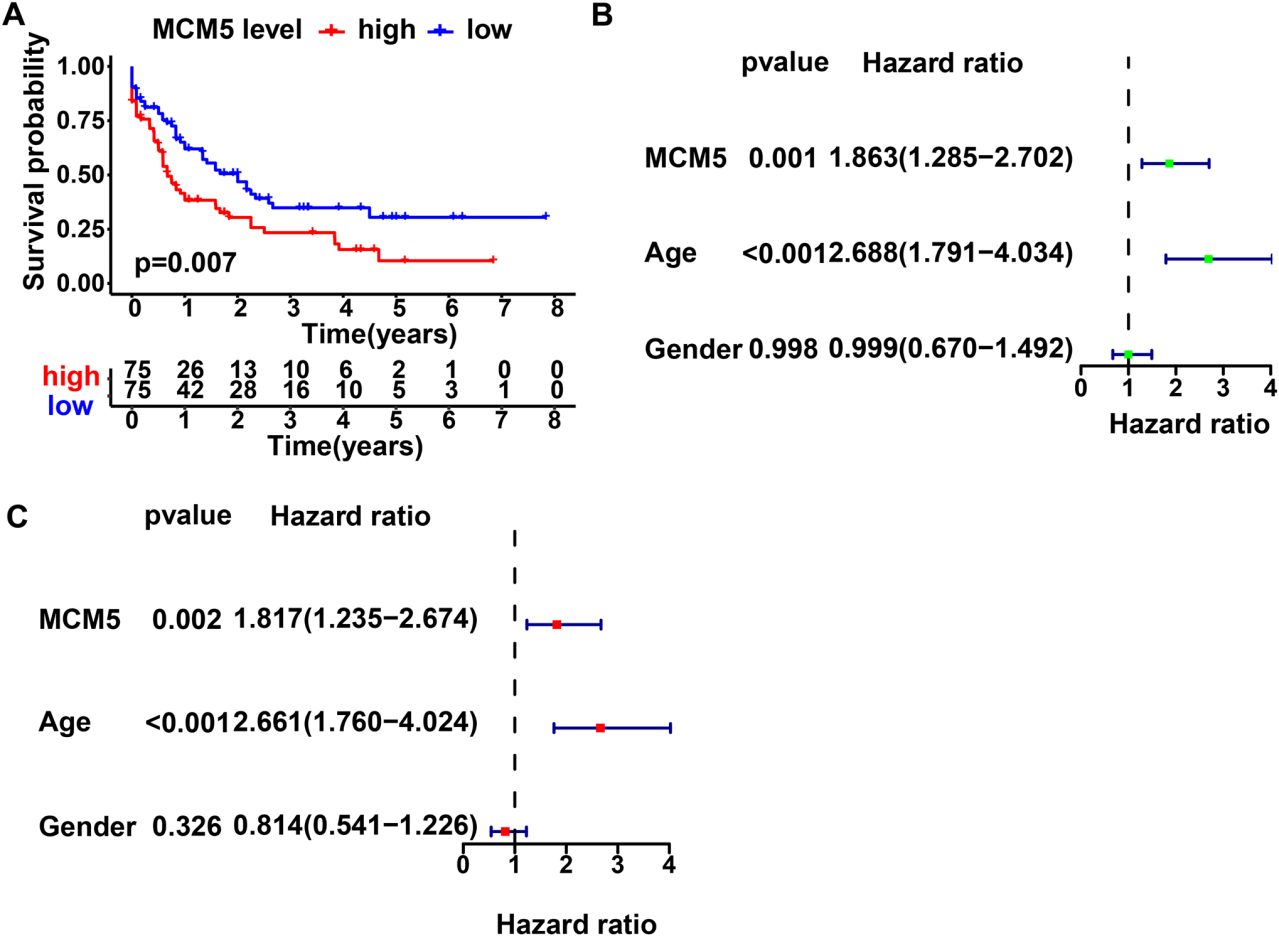
Survival analysis and Cox regression analysis of MCM5 in TCGA. **A** Survival analysis betwixt MCM5 high group and MCM5 low groups based on TCGA data. **B** Univariate Cox regression analysis and **C** multivariate Cox regression analysis screened out the individual prognostic-related element

We analyzed these MCM5 level based on the TCGA-AML. The results were consistent with those harvested from GSE38865 data. In Fig. 3, high MCM5 group had worse prognosis than low MCM5 group (Fig. 3A), and MCM5 was an independent prognostic element in AML (Fig. 3B and C).

### MCM5 Overexpression in AML

**Fig. 4:**
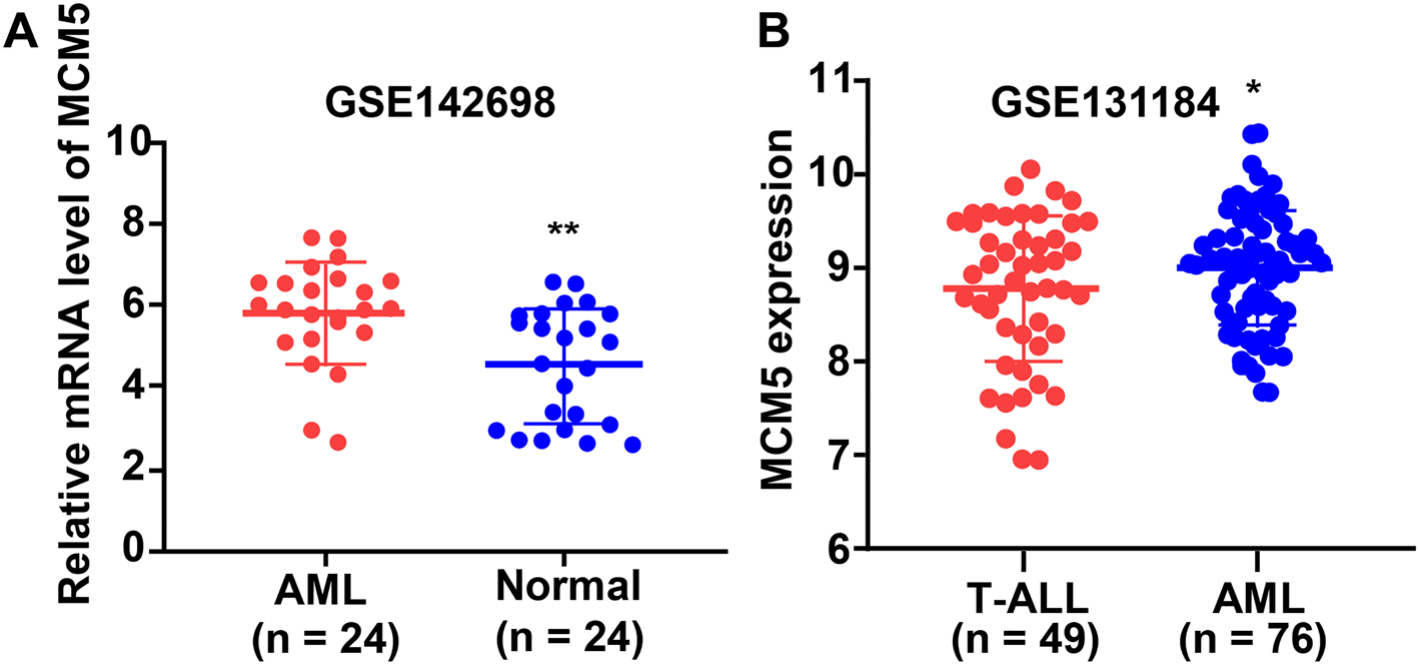
The level of MCM5. **A** The level of MCM5 in AML blood compared with healthy blood based on GSE142698. **B** MCM5 level in AML bone marrow samples compared with T-ALL bone marrow samples based on GSE131184

Then, we want to study whether MCM5 level shows difference in AML and normal samples. GSE142698 (AML = 24; normal = 24) was download from GEO database. We found an increase level of MCM5 in AML blood compared with healthy blood (Fig. 4A). We also found that an increase level in AML bone marrow samples compared with T Acute Lymphoblastic Leukemia (T-ALL) bone marrow samples (Fig. 4B) based on GSE131184 (AML = 76; T-ALL = 49).

### Differences in Clinical in AML Betwixt MCM5 High Group and MCM5 Low Groups

The differences in clinical in AML betwixt MCM5 high group and MCM5 low groups were analyzed through “ggpubr” package. There was no significant difference in the age gender, and NPM1 mutation betwixt MCM5 high group and MCM5 low groups (Fig. S1A-C).

### Different Expression and Functional Enrichment Analysis for Genes in MCM5 High Group Versus MCM5 Low Group

**Fig. 5:**
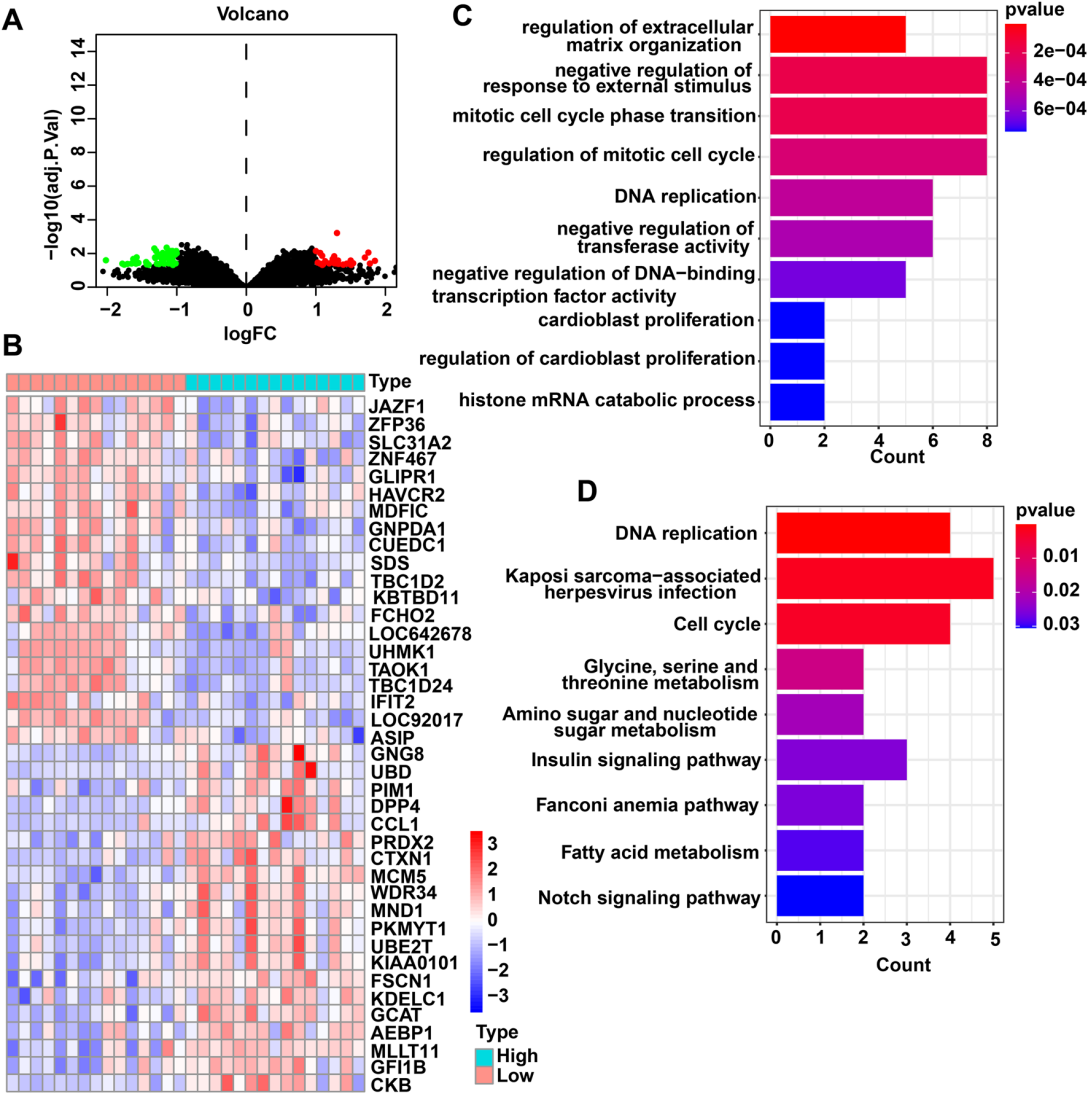
DEGs and the results of GO enrichment and KEGG analyses. **A** Volcano plot show DEGs betwixt MCM5 high group and MCM5 low groups. **B** Heatmap plot show the top 20 up-modulated DEGs and top 20 down-modulated DEGs betwixt MCM5 high group and MCM5 low groups. **C** GO result for DEGs. **D** KEGG result for DEGs

To discover the genes correlated with MCM5, we analyzed the discrepant level values betwixt MCM5 high group and MCM5 low group (adj.*p* < 0.05, (FC, log2) > 1 or < 1). There were 34 up-modulated genes and 47 down-modulated genes (Fig. 5A; Table S3). Figure 5B exhibited the top 20 genes in up-modulated and down-modulated groups. We analyzed the enriched GO terms through using the discrepantly expressed genes (DEGs). Among the biological process terms of GO, DEGs are mainly enriched in regulating extracellular matrix organization, negatively regulating response to external stimulus, phase transition of mitotic cell cycle, regulating mitotic cell cycle, and DNA replication (Fig. 5C). Showed by the KEGG analysis results, DNA replication, Kaposi sarcoma-correlated herpesvirus infection, Cell cycle, Glycine, serine and threonine metabolism, and Amino sugar and nucleotide sugar metabolism were the most enriched pathways (Fig. 5D).

### Module Screening Using PPI Network

**Fig. 6:**
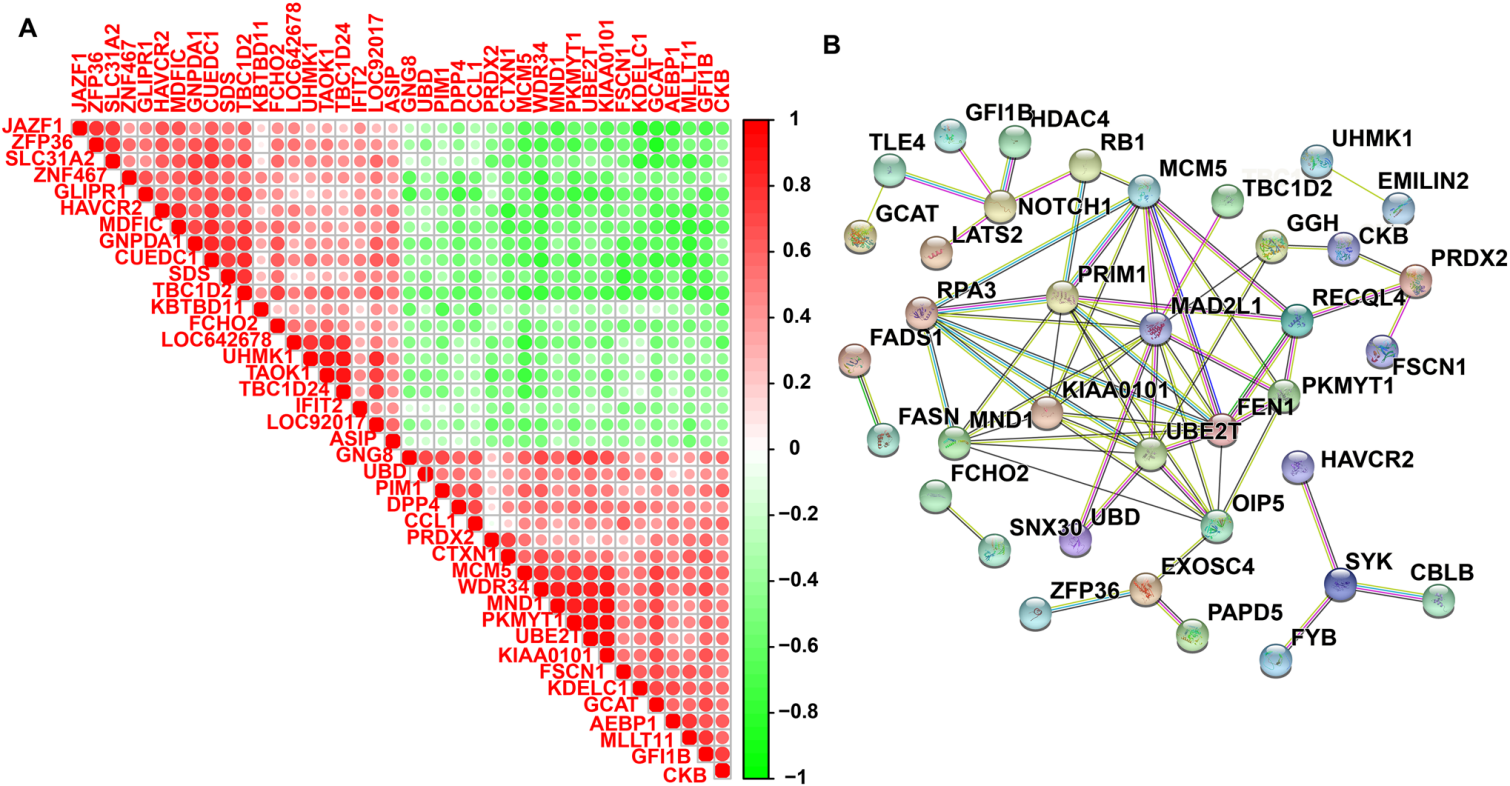
Module screening from the PPI network. **A** The correlation analysis of top 20 up-modulated DEGs and top 20 down-modulated DEGs with the Pearson correlation coefficient. **B** PPI network of DEGs

Last, the correlation analysis of the top 40 DEGs betwixt MCM5 high group and MCM5 low group was performed (Fig. 6A). We could see that MCM5 was positive correlation with KIAA0101 which also up-modulated in MCM5 high group.

KIAA0101 is a proliferating cell nuclear antigen (PCNA) -related element. KIAA0101 has been verified to be over-expressed in a variety of human malignant tumors, such as esophageal squamous cell carcinoma, lung adenocarcinoma, chronic lymphocytic leukemia, and it has been identified as an oncogene [24–28]. Protein–protein interaction (PPI) network was screened in the String database through with the 81 DEGs. All down-modulated genes and most up-modulated genes had interactions in the PPI network (Fig. 6B). In the general network MCM5 acts as a core gene. The graphical abstract is showed in Fig. S2.

## Discussion

AML is listed among the most common hematologic malignancies [29, 30]. Despite progress in the treatment of AML, it remains difficult to treat and cure, and the 5-year overall survival rate, especially in sufferers which age is over 60 years, is still very low [31–33]. The pathogenesis, diagnosis and treatment of AML are hot topics in current research.

In our research, we analyzed the mRNA and the correlative clinical information in GSE38865. We wanted to find the genes which could predict bad or good prognosis in AML. Through using Kaplan–Meier curves, Cox regression analysis, Wilcox.test or Kruskal.test, and GEPIA, we found MCM5 may the target gene which was an independent prognostic element for AML. From TCGA we also downloaded the AML-related RNA-seq data (TPM) and harvested the correlative clinical information. It was exhibited by results that MCM5 was an independent prognostic element for AML and AML sufferers in MCM5 high group had worse prognosis than that in MCM5 low group. We also found an increase level in AML blood compared with healthy blood (GSE142698) and an increase level in AML bone marrow samples compared with T-ALL bone marrow samples (GSE131184).

MCM5, as a critical cell cycle regulator and DNA replication licensing element, was expressed highly in multiple cancer tissues, such as cervical adenocarcinoma, cervical cancer, renal cell carcinoma, and laryngeal squamous cell cancer [20, 34–36]. MCM5 in thyroid cancer cells was also reported as a target of BET inhibitors. MCM5 may serve as an adverse prognostic biomarker for lung cancer[18, 37, 38]. In addition, Chen Liu even reported that compared with leukocytes, MCM5 was highly expressed in AML cell lines (KG-1a, NB4 and HL60)[12].

To find the genes correlated with MCM5, we analyzed the deferent expressed genes betwixt MCM5 high group and MCM5 low group. We found CCL1, MLLT11, and PIM1 were up-modulated in MCM5 high group. CCL1 is involved in immune-cell recruitment and, like other chemokines, is involved in nociceptive processing[39]. The chemokine CCL1 activates the AMFR-SPRY1 pathway, which facilitates differentiation of pulmonary fibroblasts into myofibroblasts and drives pulmonary fibrosis[40]. MLLT11 acted as an oncogene in multiple cancers, for example osteosarcoma[41], bladder cancer[42], and lung cancer[43]. Importantly, report exhibited that MLLT11 is a unfavorable prognostic biomarker for AML, adult normal cytogenetics AML, and adult myelodysplastic syndrome[44]. PIM1 plays a critical role in the development of many hematopoietic and non-hematopoietic malignancies, including prostate cancer and acute myeloid leukemia[45, 46]. AML with high level of PIM1 was reported to show an unfavorable prognosis. PIM1 facilitates the proliferation and inhibits apoptosis of AML cells, but also enhances the chemotactic ability of leukemia cells[47, 48].

Likewise, the GO pathways mainly enriched in regulation of extracellular matrix organization, DNA replication, mitotic cell cycle phase transition, regulation of mitotic cell cycle, mitotic cell cycle phase transition, and negative regulation of response to external stimulus. DNA replication, Kaposi sarcoma-correlated herpesvirus infection, Cell cycle, Amino sugar and nucleotide sugar metabolism, serine and threonine metabolism, and Glycine.

PPI network also revealed that MCM5 is highly linked with KIAA0101. KIAA0101 is a proliferating cell nuclear antigen (PCNA) -related element. KIAA0101 has been reported to be over-expressed in many human malignant tumors and has been identified as an oncogene[27, 49]

## Conclusions

In conclusion, we demonstrated that MCM5 was an independent prognostic element for AML. Low level of MCM5 was a good prognostic element for AML sufferers. In addition, the results of GO term enrichment, KEGG analysis and PPI network involvement in AML showed that MCM5 may regulate DNA replication and cell cycle of AML cells which provided an insight into the pathogenesis correlated with different MCM5 level. However, the exact pathophysiological role of MCM5 in AML cells has not been fully demonstrated in this study. Further investigation of the molecular mechanism of MCM5 in AML progression and more in-depth genomic studies are urgently needed.

## Supplementary Information

Below is the link to the electronic supplementary material.

Supplementary file1 (TIF 1050 kb)

Supplementary file2 (TIF 2620 kb)

Supplementary file3 (XLS 4 kb)

Supplementary file4 (DOCX 16 kb)

Supplementary file5 (XLS 6 kb)
