# Supplementary figures and images for: Enhanced MCM5 Level Predicts Bad Prognosis in Acute Myeloid Leukemia
Source: Mol Biotechnol. 2022 Dec 7;65(8):1242–52. doi: 10.1007/s12033-022-00623-9 (PMC10352173; doi:10.1007/s12033-022-00623-9)

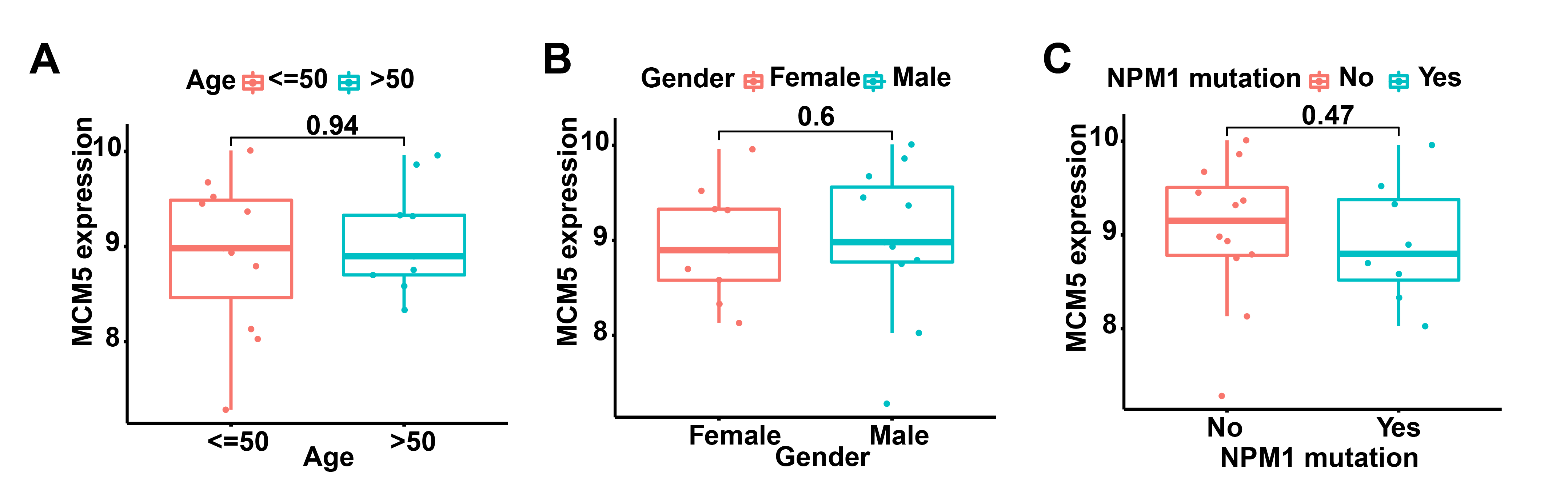

Supplement: Supplementary file 1 — Supplementary file1 (TIF 1050 kb) [file 12033_2022_623_MOESM1_ESM.tif]

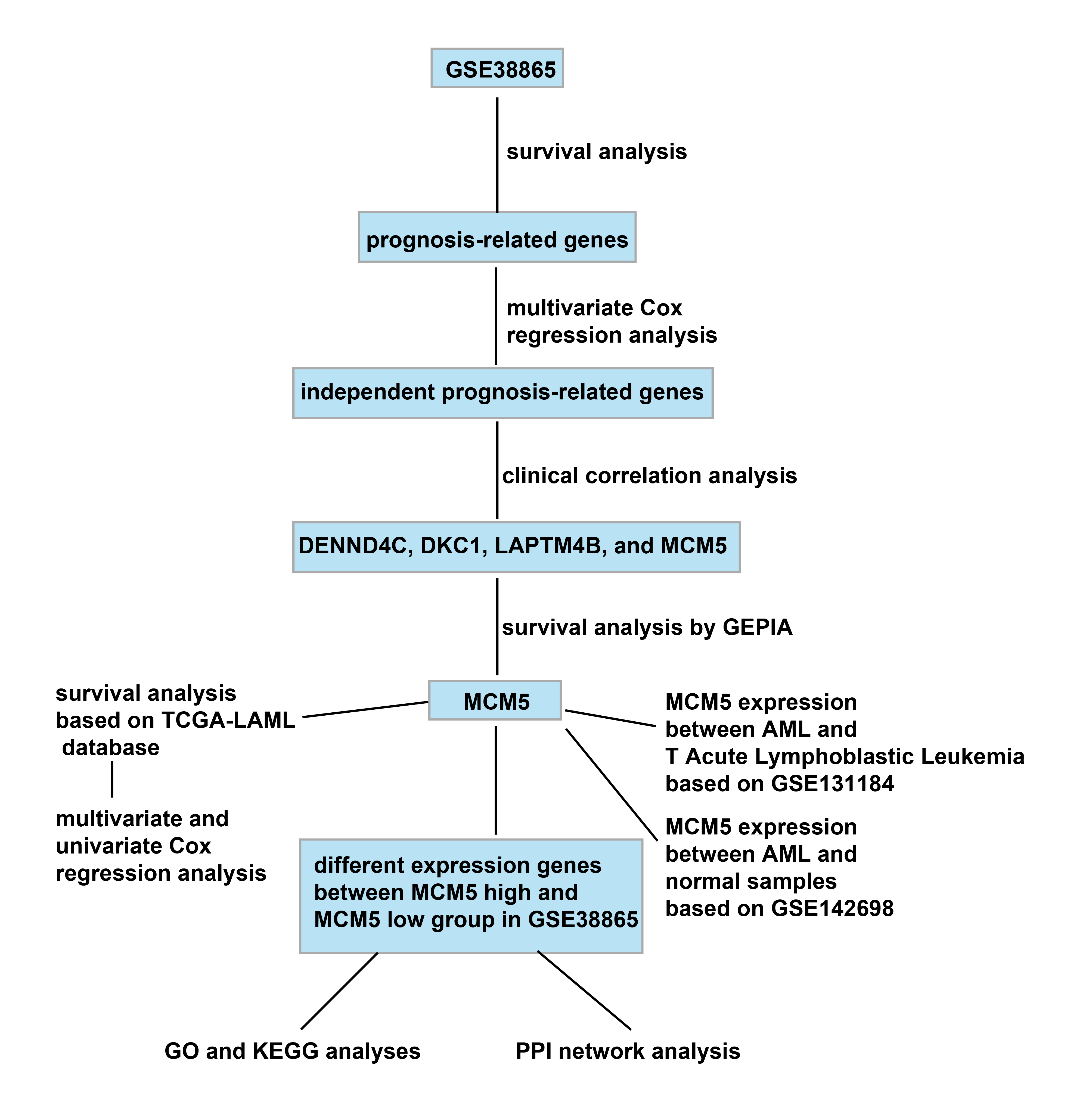

Supplement: Supplementary file 2 — Supplementary file2 (TIF 2620 kb) [file 12033_2022_623_MOESM2_ESM.tif]
