# Supplementary material for: Enhanced MCM5 Level Predicts Bad Prognosis in Acute Myeloid Leukemia
Source: Mol Biotechnol. 2022 Dec 7;65(8):1242–52. doi: 10.1007/s12033-022-00623-9 (PMC10352173; doi:10.1007/s12033-022-00623-9)
Supplement: Supplementary file 4 — Supplementary file4 (DOCX 16 kb) [file 12033_2022_623_MOESM4_ESM.docx]

Table S2 The univariate and multivariate Cox regression analysis of MCM5 in AML from GSE38865

| univariate Cox regression analysis | | | | |
| --- | --- | --- | --- | --- |
| id | HR | HR.95L | HR.95H | pvalue |
| MCM5 | 7.940111 | 1.740425 | 36.22411 | 0.007461 |
| Gender | 0.995112 | 0.298527 | 3.317118 | 0.993635 |
| Age | 1.014661 | 0.971526 | 1.059711 | 0.511399 |
| Wbc | 1.001254 | 0.992859 | 1.00972 | 0.770442 |
| NPM1 mutation | 0.857638 | 0.254304 | 2.892381 | 0.804442 |
| multivariate Cox regression analysis | | | | |
| id | HR | HR.95L | HR.95H | pvalue |
| MCM5 | 10.25121 | 1.935775 | 54.28697 | 0.006208 |
| Gender | 1.111367 | 0.120626 | 10.23938 | 0.925749 |
| Age | 1.018278 | 0.959031 | 1.081186 | 0.5537 |
| Wbc | 0.994986 | 0.985276 | 1.004792 | 0.315078 |
| NPM1 mutation | 0.730293 | 0.077346 | 6.895308 | 0.783789 |
